# Awareness, Attitudes, and Practices Toward Meningococcal B Vaccine among Pediatricians in Italy

**DOI:** 10.3390/medicina54060100

**Published:** 2018-12-03

**Authors:** Pietro Ferrara, Lucia Stromillo, Luciana Albano

**Affiliations:** Department of Experimental Medicine, University of Campania “Luigi Vanvitelli”, 5, Via Luciano Armanni, 80138 Naples, Italy; p_ferrara@alice.it (P.F.); lucy.stromillo@libero.it (L.S.)

**Keywords:** Italian Vaccine Action Plan, meningococcal B vaccine, MenB vaccine, meningitis prevention, pediatricians’ attitudes

## Abstract

*Background and objectives:* Vaccination against bacterial pathogens is decisive for preventing invasive meningococcal disease and pediatricians play a pivotal role in vaccination compliance and coverage. The aim of this study was to investigate awareness, attitude, and practices toward the vaccine against Meningococcal B serogroup (4CMenB) among a sample of Italian pediatricians. *Materials and Methods:* A cross-sectional study was carried out using an online questionnaire from March to May 2015. Three multivariate logistic regression models were built to identify factors associated with the outcomes of interest. *Results:* The data showed that 95.5% of the interviewees correctly responded about the availability of 4CMenB vaccine in Italy, while only 28.0% knew the vaccination schedule for children aged two years or under. This knowledge was significantly higher in younger pediatricians and in those who worked a higher number of hours per week. Pediatricians self-reported a positive attitude toward the utility and safety of 4CMenB vaccine. Those pediatricians with a strong positive attitude toward the utility of the vaccine, who knew the vaccination schedules for children of two years or under, and who declared a satisfactory or good knowledge about the vaccine were more likely to inform parents about its availability in Italy, recommend the vaccination, and verify patients’ vaccination status, in their daily practice. *Conclusions*: The study highlights factors that currently influence pediatricians’ practices regarding the 4CMenB vaccine. The results showed the possible actions recommended to improve physicians’ awareness and behaviors in order to improve the vaccination compliance and invasive meningococcal diseases prevention.

## 1. Introduction

Invasive meningococcal disease (IMD) is a serious public health challenge due to high mortality and morbidity in infants, children, and young adults. The global burden of the disease is caused by *Neisseria meningitidis* infection and most of the cases are caused by serogroups A, B, C, W-135, and Y. Meningococcal serotype B (MenB) and C (MenC) are responsible for the majority of cases of meningitis in Europe and in the United States [1,2,3]. In Italy, available epidemiological data show that incidence of IMDs within the age range 0 to 4 years was principally sustained by MenB *N. meningitidis*, with 62% and 61% of confirmed cases in 2015 and 2016, respectively [4].

Vaccination against the bacterial pathogen is crucial for preventing IMDs. The introduction of the vaccine against MenC and quadrivalent meningococcal conjugate vaccine (MenACWY or MCV4) provided valuable and successful strategy options for preventing infections and reducing the incidence of cases due to these serogroups [5,6]. In 2013, a vaccine containing four immunogenic components (4CMenB) was licensed in Europe against MenB, and evidence of the vaccine’s effectiveness in reducing MenB-related IMDs has been reported [7,8,9,10]. Since the European Medicines Agency (EMA) authorized its commercialization and use, Italian health authorities considered the implementation of vaccination programs through the introduction of 4CMenB vaccine targeting population at risk for MenB infection [11,12,13].

Accumulating evidence has suggested that pediatricians play a significant role in foster parental acceptability of vaccinations when they provide accurate educational messages and promote immunization compliance [14,15]. However, limited attention has been paid to investigating knowledge of healthcare providers, especially pediatricians, toward vaccination against MenB in infants, children, and young adults [15,16,17]. Therefore, the aim of the current study was to investigate awareness, attitude, and practices toward 4CMenB vaccine among Italian pediatricians.

## 2. Materials and Methods

### 2.1. Sample

A cross-sectional online survey was conducted from March to May 2015 in a sample of 700 pediatricians selected from the members of the Italian Society of Preventive and Social Pediatrics (Società Italiana di Pediatria Preventiva e Sociale, SIPPS). The SIPPS Board was contacted and informed about the nature and protocol of the study to obtain permission to submit the self-administered anonymous questionnaire. After the approval, the SIPPS Board was asked to perform a 1-in-2 systematic sampling, by selecting a random starting point and selecting other pediatricians every 2nd subject on the member list.

### 2.2. Survey Instrument

Data were collected using a self-administered semi-structured online questionnaire, initially pilot-tested on 20 pediatricians to evaluate design, clarity, and comprehensibility of the items. The research instrument, designed for capturing information from pediatricians, was divided into five sections. The first section measured demographic and professional characteristics, including age, sex, specialization, number of years in practice, years since graduation, setting of practice, number of hours worked for week, and number of patients seen for a week. The second section assessed the respondents’ knowledge about 4CMenB vaccine, including a question on its presence/absence in Italian and Regional Vaccine Action Plans (dichotomous, yes/no question) and its scheduled doses (closed-ended question). The third section consisted of 11 items that aimed to assess pediatricians’ perception toward the utility and safety of meningococcal vaccines for preventing IMDs, the effectiveness of vaccine intervention in reducing healthcare costs, the importance to introduce the 4CMenB in Vaccine Action Plans, the perception about parents’ acceptability, and the self-perceived knowledge about 4CMenB vaccine. These attitudes were measured through Likert-type scales, distributed into six items ranging from 1 (very low attitude) to 10 (extremely positive attitude), four items with options listed as ‘‘agree’’, ‘‘uncertain’’, and ‘‘disagree’’, and one item on a 5-point scale ranging from “scarce” to “excellent”. The fourth section investigated pediatricians’ practices: interviewees were asked to indicate how often, in their practice, they inform parents about the existence of the vaccine (5-point Likert scale ranging from “never” to “always”); in the subsequent questions, respondents were asked to indicate reasons whether or not they had recommended the vaccine, assessed by a list of possible reasons to motivate their practice. One more question was aimed to determine how frequently pediatricians verify patients’ vaccination status, through a five-point Likert scale ranging from “never” to “always”. Sources of information about 4CMenB vaccine were assessed in the last section of the survey, where interviewees were allowed to answer more than one of the following options: guidelines, scientific journals, educational courses, internet, colleagues, others, none. A question about the perceived needing of additional information about 4CMenB vaccine was also asked (yes/no question).

The questionnaire was delivered to all participants via professional online survey software (Google^®^ Forms, Google LLC, Mountain View, CA, USA). All pediatricians received an email inviting them to complete the survey, accessible via an embedded URL link. Non-respondents received a reminder every 3 weeks until the survey was closed. Participating pediatricians completed the web-based survey through 6 web pages that included 5 sections, always appearing in the same order. Clear preliminary statements provided information about the study, and allowed participants to confirm their own informed consent to fill the survey; involvement was voluntary and no incentives were offered to complete it. Participants were told that the questionnaire was anonymous and would take around 10 min to complete. In order to encourage participation, the survey enabled pediatricians to stop the survey at any time and to restart it as long as the survey link was active.

### 2.3. Ethics

The Ethical Committee of the authors’ institution approved the study protocol and the questionnaire, granting the approval for carrying out this research.

### 2.4. Statistical Analysis

The statistical analysis was conducted using Stata version 14 statistical software [17] and consisted of descriptive and inferential analyses. The latter was conducted in two steps through exploratory univariate analyses, using Student’s *t*-test for continuous variables and chi-square (*χ*^2^) test for categorical variables. Those variables with *p*-value ≤ 0.25 on bivariate analysis were considered for possible entry in the multivariate regressions models. Significant statistical level for *p*-values was set at 0.05 throughout the study. In order to facilitate analysis and interpretation, some variables measured on ordinal scales were dichotomized before model building.

Three separate multivariate logistic regression models were performed to determine independent characteristics associated with these outcomes: correct knowledge of the vaccination schedules for children of 2 years of age or under (Model 1), pediatricians’ positive attitude toward the strong utility of 4CMenB vaccine (Model 2), and correct practices of informing parent about the availability of 4CMenB vaccine, recommending the vaccination, and verifying patients’ vaccination status (Model 3).

In all models, the following explanatory independent variables were considered for inclusion: sex (male = 0, female = 1), age (continuous, in years), marital status (married = 1; unmarried = 0), having at least one child (yes = 1; no = 0), years of practice (continuous), years since graduation (continuous), pediatricians with more than one specialization (yes = 1; no = 0), setting of practice (primary care = 1, other = 0), number of hours worked for week (continuous), number of patients seen in a week (continuous), use of guidelines and scientific journals as source of information about 4CMenB vaccine (yes = 1; no = 0), and need of additional information about the vaccine (yes = 1; no = 0). The following variables were also included in Models 2 and 3: correct knowledge of the vaccination schedules for children under two years (yes = 1; no = 0), correct knowledge that 4CMenB vaccine is not included in Italian Vaccine Action Plan (yes = 1; no = 0), and correct knowledge that 4CMenB vaccine is not included in Regional Vaccine Action Plans (yes = 1; no = 0). Variables such as perception of utility of MCV4 and MenC vaccines (10 = 1; others = 0), perception of utility of new 4CMenB (10 = 1; others = 0), perception of safety of MCV4 and MenC vaccines (10 = 1; others = 0), perception of safety of 4CMenB (10 = 1; others = 0), and self-perceived knowledge about 4CMenB vaccine (excellent = 1; other = 0) were also included in Model 3.

According to the step-wise method for multivariate analysis, variables with a *p*-value of 0.4 and 0.2 on multivariate analysis were respectively considered for exclusion and inclusion in the final logistic regressions models. Results were reported as odds ratios (ORs) and 95% confidence intervals (CIs).

## 3. Results

A total of 700 pediatricians were selected and invited to fill out the online questionnaire; 200 agreed to participate in the study for an overall response rate of 28.6%. The main socio-demographic and professional characteristics of the study population are listed in Table 1. Approximately half the participants were men (55.0%), with a mean age of 55 years, mainly married (80.1%), and having one or more children (89.5%). More than three-quarters of interviewees (77.8%) had a 20 year-long experience in practice, whereas the proportions of employment settings were divided as 58.5% pediatricians employed in the primary care sector, 20.5% in hospitals, and the remaining 21.0% were private consultant pediatricians. The average hours worked for week were 35.2 h and the mean number of patients seen in a week 100 patients.

Regarding their knowledge of the 4CMenB vaccine, the vast majority of pediatricians (95.5%) correctly responded about its availability in Italy, but only 42.9% and 49.2% knew that the vaccine was not included in the Italian and Regional Vaccine Action Plans, respectively. The schedules for the 4CMenB vaccine for children aged two years or under were known by only 28.0% of respondents. The results of the multivariable logistic regression analysis indicated that this knowledge was higher in younger interviewees (OR = 0.78) and in those worked a higher number of hours per week (OR = 1.06) (Model 1 in Table 2).

Regarding attitudes toward the 4CMenB vaccine, 86.4% of participants agreed that the vaccine is an effective strategy for preventing IMDs and 77.0% agreed that it is an important tool for reducing the economic impact of the healthcare costs due to IMDs. Interviewees were asked about the introduction of the 4CMenB in the Vaccine Action Plans, with a 89.0% of pediatricians declaring a favorable attitude toward this possibility, even though only half of the sample (50.8%) thought that parents would accept it. The perceived utility of different meningococcal vaccines (MenACWY, MenC, and 4CMenB vaccines) showed mean values of 7.6 (SD = 2.8), 8.0 (SD = 2.9), and 8.7 (SD = 2.3) out of 10, respectively, with the 60.8% of pediatricians perceiving 4CMenB vaccine as being very useful in preventing IMDs burden caused by MenB. Results of the pediatricians’ perception of the safety of these vaccines displayed separated mean values of 8.1 (SD = 2.6), 8.6 (SD = 2.3), and 8.7 (SD = 2.1), respectively. Results of the multivariable logistic regression model indicated that a more positive attitude toward the utility of 4CMenB was observed in younger pediatricians (OR = 0.81), who had a higher number of years since graduation (OR = 1.29), in respondents who worked in the primary care sector (OR = 3.58), in those who saw a lower number of patients on a weekly basis (OR = 0.99), in those who knew that the 4CMenB vaccine was not included in Vaccine Action Plans (OR = 2.14), and in pediatricians that used guidelines and scientific journals as sources of information (OR = 2.88) (Model 2 in Table 2).

With reference to their behaviors, only 49.0% of pediatricians declared they routinely inform parents about the availability 4CMenB vaccine and 93.0% stated they recommend it, but when examining about their practice of informing parents, recommending the vaccine, and checking patients’ vaccination status, this percentage decreased to 35.5%. The results of the logistic regression analysis built to investigate the independent characteristics associated with this outcome revealed that this behavior was significantly higher in pediatricians with a strong positive attitude toward the utility of the vaccine (OR = 5.28), in those who knew the vaccination schedules for children two years old or under (OR = 3.71), and in those who self-declared a satisfactory or good knowledge about new 4CMenB (OR = 3.71) (Model 3 in Table 2).

When investigating reasons for 4CMenB vaccine recommendation, 79.0% of the interviewees specified that benefits of vaccination outweigh risks, 51.1% of the sample stated the MenB serogroup is responsible for the majority of meningitis cases in Italy, and 62.9% reported that the vaccine effectively reduces the incidence of IMDs.

In the entire sample, the main sources of information about the vaccine used by pediatricians were scientific journals (74.0%), educational courses (63.0%), and guidelines (34.0%). Finally, the overall pediatricians’ belief in their own knowledge about the vaccine was low, with only 47.5% declaring a satisfactory or good level of knowledge on the issue. A large proportion of the study population (86.9%) self-reported needing additional information about the vaccine against MenB.

## 4. Discussion

This study yielded interesting results about the awareness, attitudes, and practices in a sample of Italian pediatricians toward 4CMenB vaccine, after it was licensed by European Medicines Agency in 2013.

As a first step forward in the study of pediatrician knowledge, despite almost all interviewees (95.5%) being aware about the availability of the vaccine against MenB in Italy, a relevant proportion of them answered that the 4CMenB vaccine was included in the Italian Vaccine Action Plan (as in regional plans) in force at the time when the survey was conducted (Piano Nazionale della Prevenzione Vaccinale 2012–2014), whereas it was not included until the Vaccine Action Plan adopted in 2017 [18,19]. However, 57.1% and of 50.8% of respondents thought, respectively, that the 4CMenB vaccine was included in Italian and Regional Vaccine Action Plans not only highlighted a low level of knowledge on that specific item, but could even be considered as a proxy of the low level of pediatrician understanding of the immunization plans.

Based on the suggested scheme for the administration of the 4CMenB vaccine [20], this study was designed to investigate pediatrician knowledge of the vaccine schedules for children two years old or younger. Self-reported surveys displayed a worrisome extremely low proportion (28.0%) of interviewees knowing these schemes. The multivariate analysis suggested the most knowledgeable were younger pediatricians and respondents with a higher number of hours worked per week. These results can be easily explained as long-term practice increases the self-level of expertise in healthcare workers [15], and younger age of physicians is related to higher compliance in continuing medical education and in updating about medical science [21].

Regarding attitudes, a large part of the sample supported the 4CMenB vaccination itself as an important public health tool for reducing the costs of the impact of health care caused by IMDs. The majority of pediatricians (89%) responded favorably to the introduction of vaccine in the plans for the immunization of infants, children, and young adults, although just slightly more than half (55%) perceived the vaccine as safe. Similar results were found in other studies, where pediatricians appeared to have a positive attitude toward the 4CMenB, but declared themselves worried about the timing and spacing of closer vaccine doses [22,23].

The average respondents’ perception of the utility of vaccines for preventing IMDs, measured on aa 10-point Likert scale, scored highly for the 4CMenB vaccine (8.7 out of 10) when compared with MenACWY and MenC vaccines (7.6 and 8.0, respectively). This may be due to the role of MenB in causing the higher incidence of B meningitis in the infant population [4,13]. Different investigated factors appeared significantly associated, after regression analysis, with pediatrician attitude of the utility of the 4CMenB vaccine, confirming data from previous surveys [16,22]. Among the principal associated factors, the most appropriate sources of information played a key role, with a higher proportion of pediatricians declaring continuing their medical education, on the subject of interest, through guidelines and scientific journals.

Results regarding the behaviors were also notable. As this study was conducted during the first period after the 4CMenB vaccine distribution, only half of the interviewed admitted informing parents about its availability. Again, although higher compliance is probably not yet a realistic goal, almost all pediatricians (93.0%) stated they recommend the vaccination itself, confirming the results of Memeli’s research [22]. This study adds further data to this area, also investigating reasons for the vaccine: pediatricians mostly specified that they recommended 4CMenB because it has more benefits than risks (79.0%), is effective in reducing IMD cases (62.9%), and MenB is the most widespread serogroup in Italy (51.1%).

Briefly, data from this cross-sectional survey determined the general levels of knowledge and practices of Italian pediatricians after the introduction of the 4CMenB vaccine. Results highlighted a gap in pediatricians’ practice that must be considered and improved. Evidence suggested the importance of appropriate educational information addressed to parents on the safety and the effectiveness of the vaccination, with the aim of increasing vaccination coverage rates, since pediatricians play a significant powerful role in increasing compliance, particularly when vaccines are recommended [14,24]. This matter takes on particular importance in the Italian framework, where there have been decreases in vaccination compliance, with worrisome consequences on individual and public health [25,26]. Pediatricians’ comprehensive knowledge represents a prerequisite for motivating patients (or their parents) to undertake necessary vaccinations. Findings from this research emphasized that, regarding the MenB vaccine, this knowledge is far from being satisfactory and more educational interventions are needed to enhance pediatricians’ role in reducing IMD cases through correct immunizations programs.

Vaccines are the most cost-effective intervention available so far in health care and vaccination programs lead to an appreciable reduction in morbidity and mortality, and links population health to economic well-being [27]. Yet, regarding IMD, evidence suggests that the impact of the introduction of routine vaccines into an immunization program is a successful public health intervention [5,6,7]. The insertion of 4CMenB vaccine amongst the new vaccinations added to the current Italian Vaccine Action Plan 2017–2019 aims to markedly reduce meningitis cases amongst children [18,19]. Pediatricians—as all healthcare professionals—are required to promote patients’ adherence to immunization programs.

Some potential limitations must be considered in interpreting the findings of this survey. The first is the cross-sectional design of the study, which prevents the establishment of causal and temporal directionalities in the relationship between the dependent and independent variables. The second limitation is that the survey used self-reported information, which may overestimate or underestimate the actual pediatricians’ awareness, attitudes, and practices toward B Meningococcal vaccine, although administering an online written survey with questions focused on topic areas is supposed to minimize this risk. Thirdly, a possible non-response bias must be considered, since it was difficult to assess characteristics of pediatricians who chose to not participate in the survey. Again, the sample may not be representative of the whole Italian population of pediatrics. Lastly, in discussing this result, this survey was submitted before that the vaccination was widely available in Italy, since its recommendation might differ in each region of the country, where the vaccine may be not offered to the population [28].

Despite these limitations, the strengths of this study were that the sample was acceptable and properly selected, as well as the survey’s results provided useful relevant information about awareness, attitudes, and practices toward B Meningococcal vaccine in Italian pediatricians.

## 5. Conclusions

In conclusion, it is important that pediatricians, as well as all clinicians, fully understand the 4CMenB vaccine and the vaccination schedule. Education programs play a fundamental role in improving pediatrician knowledge in order to improve vaccination compliance and IMD prevention.

## Figures and Tables

**Table 1:** Selected characteristics of the study population (*n* = 200).

Characteristic *	Value	
Sex, *n* (%)		
Male	110	55.0
Female	90	45.0
Age, mean ± SD (range), years	54.7 ± 9.0 (27–73)	
Children, *n* (%)		
No	21	10.5
One or more	179	89.5
Years since graduation, mean ± SD (range)	28.3 ± 9.3 (2–48)	
Number of specializations, *n* (%)		
1	153	76.5
>1	47	23.5
Years of activity, mean ± SD (range)	23.4 ± 9.4 (2–46)	
Setting of practice, *n* (%)		
Primary care	117	58.5
Hospital	41	20.5
Private consultant	42	21.0
Number of hours worked per week, mean ± SD (range)	35.2 ± 10.4 (6–60)	
Number of patients seen per week, mean ± SD (range)	99.2 ± 59.5(14–280)	

* Number for some items may not add up to total number of study population due to missing value.

**Table 2:** Multivariate regression models results.

Variable	OR	SE	95% CI	*p*-Value
**Model 1.** Outcome: Correct knowledge of 4CMenB vaccination schedule for children aged 2 years or under Log likelihood = −57.23, *χ*^2^ = 20.59 (6 df), *p* = 0.002				
Age (continuous, in years)	0.78	0.09	0.63–0.97	0.03
Number of hours worked per week (continuous)	1.06	0.03	1.01–1.12	0.03
Marital status Years since graduation (continuous) Sex (male) Number of patients seen per week (continuous)	0.38 1.19 0.47 1.01	0.22 0.13 0.25 0.00	0.12–1.17 0.96–1.48 0.16–1.36 1.00–1.01	0.09 0.10 0.16 0.16
**Model 2.** Outcome: Positive attitude toward strong utility of 4CMenB vaccine Log likelihood = −97.51, *χ*^2^ = 33.97 (8 df), *p* < 0.0001				
Years since graduation (continuous)	1.29	0.10	1.10–1.51	0.002
Setting of practice (primary care)	3.58	1.55	1.54–8.35	0.003
Age (continuous, in years)	0.81	0.07	0.69–0.96	0.01
Number of patients seen per week (continuous)	0.99	0.00	0.98–0.99	0.01
Correct knowledge that 4CMenB is not included in Regional Action Plans	2.14	0.79	1.04–4.40	0.04
Continuing education through guidelines and scientific journals	2.88	1.51	1.04–8.02	0.04
Correct knowledge of vaccination schedule for children under 2 years	2.58	1.54	0.80–8.29	0.11
Children (one or more)	2.08	1.46	0.53–8.20	0.30
**Model 3.** Outcome: Correct practices of informing parents about the availability of 4CMenB vaccine, recommending the vaccination, and verifying patients’ vaccination status Log likelihood = −89.55, *χ*^2^ = 43.72 (4 df), *p* < 0.0001				
Positive attitude toward the strong utility of the 4CMenB vaccine	5.28	2.24	2.30–12.14	<0.001
Confidence of a good personal knowledge about 4CMenB vaccine	4.02	1.53	1.91–8.46	<0.001
Correct knowledge of vaccination schedule for children under 2 years	3.71	1.88	1.38–9.99	0.009
Setting of practice (primary care)	2.10	0.85	0.95–4.66	0.06

4CMenB, vaccine against B meningococcal serogroup; CI, confidence interval; OR, odds ratio; SE, standard error; df, degrees of freedom.
